# Short Editorial: Hypertension in Special Populations: An Epidemiological Challenge

**DOI:** 10.5935/abc.20190180

**Published:** 2019-09

**Authors:** Rui Póvoa

**Affiliations:** Universidade Federal de São Paulo, São Paulo, SP - Brazil

**Keywords:** Hypertension/epidemiology, Hypertension/prevention & control, African Continental Ancestry Group/genetics, Risk Factors, Tobacco Use Disorder, Alcoholism

Arterial hypertension (AH) is the most prevalent chronic disease worldwide and the main risk factor for most cardio-cerebrovascular diseases.^1^ The true prevalence in Brazil is still unknown, and the available data are from the Vigitel Study, where the information is obtained by telephone contact. The prevalence of hypertension in Brazil is estimated at around 31% in adult individuals.^2^ In recent data from the Vigitel Study, the prevalence was 25.7% of the adult Brazilian population.^3^ Knowledge of the real prevalence and geographic distribution is not only important for prevention and treatment measures, but also contribute to the knowledge of the genesis of the disease.

In some populations, particularly individuals of African descent, AH has its own characteristics, including prevalence, therapeutic response and severity.^4,5^ The multifactorial aspect of AH is only understood when assessing special populations considering their own habitats and habits, as in the case of *quilombolas*, where individuals with African ancestry still retain some genetic and cultural characteristics of the African origin.^6^ The analysis in this context is important, since we can detect aspects inherent to factors related to AH development.

In this study,^6^ the prevalence of hypertension in the *quilombola* communities of Sergipe was 26%, with the authors reporting that the mean value in the state is much lower (20.4%).^7^ However, the values are very similar to those found in the Vigitel Study, which attempts to represent the Brazilian population. Regarding the risk factors for AH, in this population with a certain degree of vulnerability, the study disclosed inadequate lifestyle habits, especially physical inactivity, smoking and alcohol consumption. The quantification of salt in the diet was not accurate, as more complex tests are needed to determine the values, and the authors justify the fact by the study's own limitation.^8^

Knowledge of these risk factors for both hypertension and cardiovascular events is important for the planning of health actions in these at-risk populations. This study^6^ has a very significant epidemiological value, as it allows social considerations and extrapolation to other *quilombola* communities, so that health team interventions can achieve a better cardiovascular prevention.
